# Accuracy of commercial kits and published primer pairs for the detection of periodontopathogens

**DOI:** 10.1007/s00784-016-1748-9

**Published:** 2016-03-29

**Authors:** Elisabeth Santigli, Eva Leitner, Gernot Wimmer, Harald H. Kessler, Gebhard Feierl, Martin Grube, Katharina Eberhard, Barbara Klug

**Affiliations:** 1 Division of Orthodontics and Maxillofacial Orthopedics, Department of Dentistry and Maxillofacial Surgery, Medical University of Graz, Billrothgasse 4, A-8010 Graz, Austria; 2 Bacteriology and Mycology Laboratory, Institute of Hygiene, Microbiology and Environmental Medicine, Medical University of Graz, Universitätsplatz 4, 8010 Graz, Austria; 3 Division of Prosthodontics, Restaurative Dentistry, Periodontology and Implantology, Department of Dentistry and Maxillofacial Orthopedics, Medical University of Graz, Billrothgasse 4, 8010 Graz, Austria; 4 Research Unit Molecular Diagnostics, Institute of Hygiene, Microbiology and Environmental Medicine, Medical University of Graz, Universitätsplatz 4, 8010 Graz, Austria; 5 Institute of Plant Sciences, University of Graz, Holteigasse 6, 8010 Graz, Austria; 6 Center of Medical Research, Medical University of Graz, Stiftingtalstraße 24/1, 8010 Graz, Austria

**Keywords:** *Aggregatibacter actinomycetemcomitans*, *Campylobacter rectus/showae*, *Eikenella corrodens*, *Fusobacterium nucleatum*, Microbiological diagnosis, Mock community, Molecular diagnostics, Oral biofilm, *Parvimonas micra*, Periodontal diagnostics, *Prevotella intermedia*, *Prevotella nigrescens*, *Streptococcus mitis*, *Streptococcus mutans*, *Veillonella parvula*

## Abstract

**Objectives:**

Despite the input of microbiome research, a group of 20 bacteria continues to be the focus of periodontal diagnostics and therapy. The aim of this study was to compare three commercial kits and laboratory-developed primer pairs for effectiveness in detecting such periodontopathogens.

**Materials and methods:**

Fourteen bacterial mock communities, consisting of 16 randomly assembled bacterial strains, were used as reference standard for testing kits and primers. Extracted DNA from mock communities was analyzed by PCR in-house with specific primers and forwarded for analysis to the manufacturer’s laboratory of each of the following kits: ParoCheck®Kit 20, micro-IDent®plus11, and Carpegen® Perio Diagnostik.

**Results:**

The kits accurately detected *Fusobacterium nucleatum*, *Prevotella intermedia*/*Prevotella nigrescens*, *Parvimonas micra*, *Aggregatibacter actinomycetemcomitans*, *Campylobacter rectus/showae*, *Streptococcus mitis*, *Streptococcus mutans*, and *Veillonella parvula*. The in-house primers for *F.nucleatum* were highly specific to subtypes of the respective periopathogen. Other primers repeatedly detected oral pathogens not present in the mock communities, indicating reduced specificity.

**Conclusions:**

The commercial kits used in this study are reliable tools to support periodontal diagnostics. Whereas the detection profile of the kits is fixed at a general specificity level, the design of primers can be adjusted to differentiate between highly specific strains. In-house primers are more error-prone. Bacterial mock communities can be established as a reference standard for any similar testing.

**Clinical relevance:**

The tested kits render good results with selected bacterial species. Primers appear to be less useful for routine clinical diagnostics and of limited applicability in research. Basic information about the periodontopathogens identified in this study supports clinical decision-making.

## Introduction

Fighting periodontal diseases is one of the major goals of oral health care, as such diseases affect more than two thirds of the world population [1]. Periodontitis is a complex disease, with multiple causal factors simultaneously and interactively modulating the initiation, progression, and severity of its course [2]. Four main risk factors are recognized today, the foremost being dental and subgingival microbiota (referred to as “oral microbiota” or “oral biofilm”), in addition to individual genetic variability, lifestyle, and systemic factors [3–6]. Prime attention has previously been given to identifying the specific periodontopathic microorganisms, as both the onset and the characteristics of periodontal diseases are closely related to changes in the physiological oral habitat. When focusing on changes in this microbial community, different molecular methods can be used to detect oral pathogens related to periodontal disease [7–10]. Recently, laborious and time-consuming culture assays have been replaced by quick and easy molecular methods. Techniques such as fluorescence in situ hybridization and confocal laser scanning microscopy, DNA-DNA hybridization, PCR, real-time PCR, and, more recently, next-generation sequencing have not only accelerated but also expanded the quest for unknown periodontopathogens [11–14]. Research results continue to broaden the picture of periodontal disease by applying novel methods to identify new bacterial species. The information gained from such studies is quite complex and standardized methods of data analyses are still being developed [15, 16].

A well-defined group of bacteria continues to be the focus of periodontal diagnostics and therapy. A specific bacterial composition of periodontopathic biofilm was first described in detail by Socransky and Haffajee. Five bacteria complexes are thus associated with periodontal disease to a varying extent, serving as targets or biomarkers for clinical research [17]. The red complex, which contains the bacteria that are most strongly related to periodontitis, has been studied extensively [18–21]. Yet, less has been reported about the relationship between periodontal disease and bacteria belonging to other complexes. In the last two decades, several commercial kits, based on various molecular technologies such as DNA-DNA-hybridization and real-time PCR, have been developed for quick and accurate identification of such bacteria. Such kits are designed to support dentists in clinical decision-making. Not only do the kits indicate the presence of certain periodontopathogens but they also propose antimicrobial therapy options.

The use of mock communities as a reference standard has been previously shown to facilitate standardization, analysis and interpretation of microbiome data [22, 23]. Bacterial mock communities, consisting of a custom-selected variety of bacterial species, can also be employed to test the accuracy of primers and probes used in kits. For the present study, such microbial mock communities were randomly composed from a pool of 16 different bacterial species that represent key periodontal pathogens and other oral or human-associated bacteria. These mock communities were used to test the capability of accurately identifying bacteria, as shown by (1) three commercial kits: ParoCheck®Kit20 (Greiner Bio-One GmbH, Frickenhausen, Germany), micro-IDent®plus11 (Hain Lifescience GmbH, Nehren, Germany), Carpegen® Perio Diagnostik (Carpegen GmbH, Münster, Germany) and (2) eleven previously published PCR primer pairs [7, 24–27].

## Methods

### Mock communities used as reference standard in this study

The bacterial strains for the mock communities were selected from reference strains based on the following criteria: (1) availability at the microbiological laboratory of the Institute of Hygiene, Microbiology and Environmental Medicine (IHMEM), Medical University of Graz; (2) suitability in terms of corresponding with the detection profile of the three commercial kits and the primers while roughly covering half of the species potentially identified by the kits (ParoCheck®Kit 20: 9 of 20; micro-IDent®plus11: 6 of 11 and Carpegen® Perio Diagnostik: 3 of 6); (3) control strains reflecting commensals or contaminants of the oral cavity. Strains were included from the American Type Culture Collection (ATCC, Manassas, USA), Belgian Coordinated Collection of Microorganisms (LMG, University of Ghent, Belgium) and clinical isolates identified through 16S rRNA gene sequencing at the Institute of Hygiene, Microbiology and Environmental Medicine, Medical University of Graz. Within the selected pool of bacteria, more than half belonged to the microbial complexes described by Socransky [3]: *Campylobacter rectus* (LMG 7612), *Fusobacterium nucleatum* subsp. *nucleatum* (ATCC 25586), *Prevotella intermedia*, *Peptostreptococcus micros* (synonymous with *Parvimonas micra*) (ATCC 33270), *Streptococcus mitis, Eikenella corrodens*, *Aggregatibacter actinomycetemcomitans* serotype b (ATCC 43718), *Capnocytophaga canimorsus*, and *Veillonella parvula.* To test for specificity, additional control strains were added to the mock communities, including *Aggregatibacter aphrophilus*, *Campylobacter coli* (LMG 9220), *Escherichia coli* (ATCC 25922), *Neisseria subflava*, *Prevotella denticola*, *Porphyromonas somerae*, and *Streptococcus mutans* (all information on strains is listed in Table 1).

**Table 1:** Laboratory strains used in the mock communities

Strain ID	Strain	Source
A	*Fusobacterium nucleatum* subsp *nucleatum*	ATCC 25586
B	*Prevotella intermedia/ nigrescens *resp	MUGS
C	*Campylobacter rectus*	LMG 7612
D	*Aggregatibacter actinomycetemcomitans* Serotype b	ATCC 43718
E	*Peptostreptococcus micros* (syn *Parvimonas micra)*	ATCC 33270
F	*Streptococcus mutans*	MUGS
G	*Veillonella parvula*	MUGS
H	*Neisseria subflava*	MUGS
I	*Eikenella corrodens*	MUGS
J	*Streptococcus mitis*	MUGS
K	*Aggregatibacter aphrophilus*	MUGS
L	*Prevotella denticola*	MUGS
M	*Capnocytophaga canimorsus*	MUGS
N	*Campylobacter coli*	LMG 9220
O	*Porphyromonas somerae*	MUGS
P	*Escherichia coli*	ATCC 25922

*ATCC* American Type Culture Collection (Manassas, USA), *LMG* Belgian Coordinated Collection of Microorganisms (University of Ghent, Belgium), *MUGS* Graz 16S rRNA Gene Sequencing Collection (Medical University of Graz, Austria)

### Preparation of bacterial suspensions for mock community randomization

**Fig. 1:**
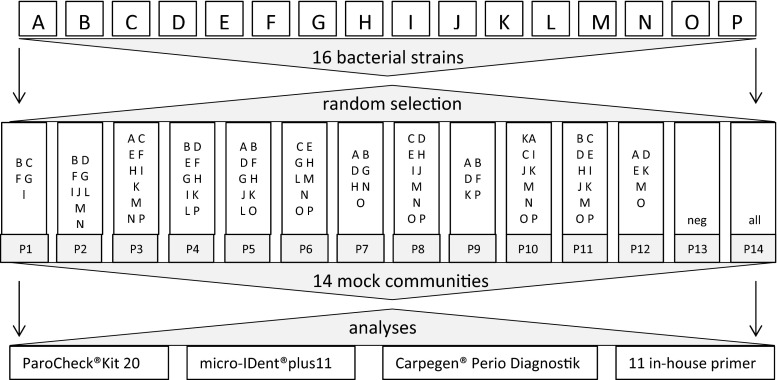
Study design and mock community composition by random selection. (Each bacterial strain present in six to nine samples)

Twelve bacterial mock community compositions were assessed by a random generator. Each strain was randomly added to four samples and excluded from four other ones. For the remaining four samples a 50/50 probability decided over addition of this strain. Additionally, one negative control (DNA-free water) and one positive control (all bacteria) were prepared, resulting in a total of 14 samples (P1 - P14 in Fig. 1). To mix bacteria in defined numbers according to bacterial culture, stock suspensions of each species were prepared in DNA-free water with turbidity equivalent to a McFarland standard of 0.5 (corresponding to 1.5 × 10 + E08 CFU/ml). The stock solutions were then used to compose 12 defined mock communities by random selection, with each bacterial species subsequently assigned between six and nine times. The final concentration of each bacterium in the mock was 7.5 × 10 + E06 CFU/ml. Depending on the number of bacteria in the mock, concentrations varied between 3.0 × 10 + E07 CFU/ml and 1.2 × 10 + E08 CFU/ml.

### DNA extraction

Prior to DNA extraction, bacterial suspensions were pretreated by mixing 180 μl of MagNA Pure Bacteria Lysis Buffer (Roche Applied Science, Penzberg, Germany), 20 μl of proteinase K solution (20 g/l), and 200 μl of the bacterial suspension. The mixture was incubated at 65 °C for 10 min and at 95 °C for another 10 min. After transferring 400 μl of the suspension to the MagNA Pure Compact sample tube, the DNA was extracted through a fully automated procedure using the MagNA Pure Compact instrument (Roche) with the MagNA Pure Compact Nucleic Acid Isolation Kit I (Roche). The DNA bacterium purification protocol was chosen with an elution volume of 50 μl. The extraction was performed in duplicate, and eluates were pooled to a final volume of 100 μl. These comparable eluates were stored at ms 70° C pending analysis.

### Commercial kits

Three commercial kits were applied to test the accuracy in identifying selected bacterial species assigned to the 14 mock communities as mentioned above. Referred to below also as “samples,” the DNA extracted from the mock communities was forwarded for analysis to the manufacturer’s laboratory of each of the following *in vitro* diagnostics (IVD) kits carrying the Conformité Européene (CE) label: ParoCheck®Kit 20 (Greiner Bio-One, Frickenhausen, Germany), micro-IDent®plus11 (Hain Lifescience GmbH, Nehren, Germany) and Carpegen®Perio Diagnostik (Carpegen, Münster, Germany). ParoCheck®Kit 20, based on DNA chip technology, is used in the analysis of 20 periodontopathogens (detection limit of 10 + E03 bacterial genomes). micro-IDent®plus11, based on a DNA hybridization technology known as DNA-STRIP®, supports the detection of 11 periodontopathogens (detection limit of 10 + E03 bacterial counts for *Aggregatibacter actinomycetemcomitans* and 10 + E04 for the other ten bacteria). Carpegen® Perio Diagnostik is a real-time PCR kit for identifying six periodontopathogens (detection limit of 2.5 × 10 + E02 bacterial counts). A summary of the bacteria detected by the kits and their assignment to the microbial complexes described by Socransky [3] is given in Table 2.

**Table 2:**
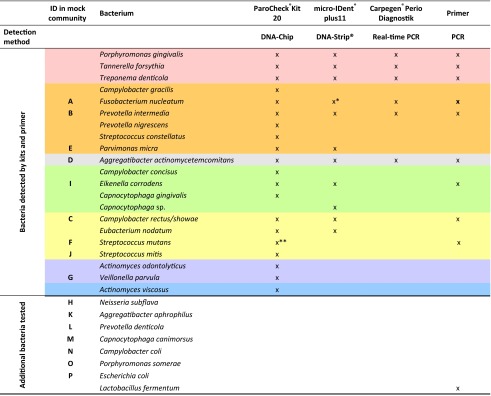
Bacteria in mock communities and detection profiles of test kits and published PCR primers

Colors assigned according to microbial complexes defined by Socransky et al. (1998)

^a^Including *Fusobacterium periodonticum*

^b^Included in *Streptococcus gordonii* group

### PCR primers

Previously published primer pairs (referred to below as “primers”) were applied in-house to identify the selected bacteria in the mock communities. For PCR, Illustra™ puReTaq Ready-To-Go PCR Beads (GE Healthcare) were used with 1–5 μl template DNA and 1–2 μl primer (10 pmol/μl) per 25 μl as described in the manual. In cases where no signals resulted with the Illustra beads, PCR amplification was repeated with Phusion polymerase (Biozym) using 5 μl template DNA and 1.5 μl primer per 25 μl PCR reaction. Singleplex PCR was performed in all cases according to protocols found in original publications (Table 3).

**Table 3:** Primer details

Primer name	Sequence 5’-3’	Bacterium	Base position (amplicon length in bp)	Reference
PoGifw^a^	AGG CAG CTT GCC ATA CTG CG	*Porphyromonas gingivalis*	729-1,132 (404)	Slots et al. [24]
PoGirev^a^	ACT GTT AGC AAC TAC CGA TGT			
TaFofw^a^	GCG TAT GTA ACC TGC CCG CA	*Tannerella forsythia*	120-760 (641)	Slots et al. [24]
TaForev^a^	TGC TTC AGT GTC AGT TAT ACC T			
TreDefw^a^	TAA TAC CGA ATG TGC TCA TTT ACA T	*Treponema denticola*	193-508 (316)	Slots et al. [24]
TreDerev^a^	TCA AAG AAG CAT TCC CTC TTC TTC TTA			
Fv35-F1	ATA ATG TGG GTG AAA TAA	*Fusobacterium nucleatum subsp. vincentii*	not available (208)	Shin et al. [27]
Fv35-R1	CCC AAG GAA AAT ACT AA			
Fs17-F14	GAT GAG GAT GAA AAG AAA CAA AGT A	*Fusobacterium nucleatum subsp. fusiforme*	not available (393)	Shin et al. [27]
Fs17-R14	CCA TTG AGA AGG GCT ATT GAC			
PrInfw^a^	TTT GTT GGG GAG TAA AGC GGG	*Prevotella intermedia*	458-1,032 (404)	Ashimoto et al. [25]
PrInrev^a^	TCA ACA TCT CTG TAT CCT GCG T			
AgAcfw^a^	AAA CCC ATC TCT GAG TTC TTC TTC	*Aggregatibacter actinomycetemcomitans*	478-1,034 (557)	Ashimoto et al. [25]
AgAcrev^a^	ATG CCA ACT TGA CGT TAA AT			
EiCofw^a^	CGA TTA GCT GTT GGG CAA CTT	*Eikenella corrodens*	not available (410)	Furcht et al. [7]
EiCorev^a^	ACC CTC TGT ACC GAC CAT TGT AT			
CaRefw^a^	TTT CGG AGC GTA AAC TCC TTT TC	*Campylobacter rectus*	415-1,012 (598)	Slots et al. [24]
CaRerev^a^	TTT CTG CAA GCA GAC ACT CTT			
Sm479F	TCG CGA AAA AGA TAA ACA AAC A	*Streptococcus mutans*	599-1,077 (478)	Chen et al. [26]
Sm479R	GCC CCT TCA CAG TTG GTT AG			
LF1	AAT ACC GCA TTA CAA CTT TG	*Lactobacillus fermentum*	196-529 (337)	(Dickson et al., 2005)
LF2	GGT TAA ATA CCG TCA ACG TA			

^a^Names assigned in this study

### Statistical analyses

All statistical analyses were performed using SPSS version 21.0 (SPSS Inc., Chicago IL, USA). Clinical sensitivity and specificity were calculated on the basis of contingency tables. Cohen’s Kappa coefficient, a statistical measure of inter-rater agreement, was used to calculate the concordance of positive and negative test results. Complete agreement corresponds to κ = 1, while lack of agreement (i.e., purely random coincidences of rates) corresponds to κ = 0. A *p* value < 0.05 was considered statistically significant.

## Results

All commercial kits performed well in the detection of the following periodontopathogens: *F. nucleatum, P. micra, A. actinomycetemcomitans* serotype b*, C. rectus/showae, S. mutans, S. mitis* group, and *V. parvula. P. intermedia* was not, however, detected by any of the three commercial kits; hence, this laboratory strain was retested and identified as *P. nigrescens*. After redefinition, negative results for *P. intermedia* were accurately obtained with all three kits. Two false positives remained, when PCR primers were applied to identify *P. intermedia* and then *P. nigrescens. E. corroden*s as part of the mock communities was falsely identified positively three times out of seven by both the PCR primers and ParoCheck®Kit 20. The primer pairs tested in this study showed the poorest performance of all test systems (Table 4).

**Table 4:**
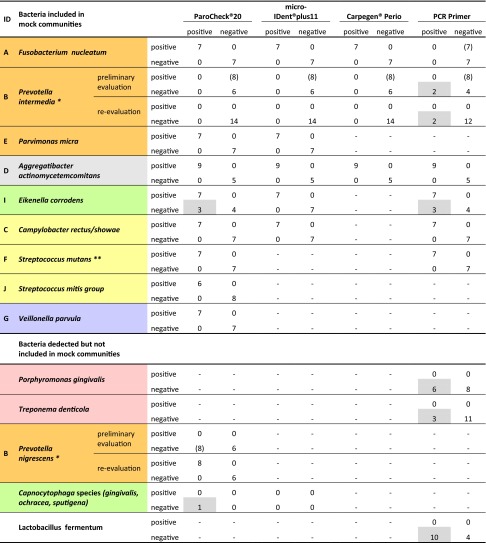
Detection of selected bacteria in 14 mock communities—three test kits and PCR primer on the test bench

^a^Re-evaluation revealed *Prevotella nigrescens* instead of *Prevotella intermedia* in the mock community

^b^Included in *Streptococcus gordonii* group

### ParoCheck®Kit 20

When the mock communities were used to test the *ParoCheck®Kit20* DNA chip, eight out of nine periodontopathogens were identified accurately: *A. Actinomycetemcomitans* serotype b was correctly identified in all cases. *F. nucleatum*, *P. nigrescens*, *P. micra*, all representatives of the orange complex and representatives of the yellow complex, such as *C. rectus/showae*, *S. mutans* as part of the *Streptococcus gordonii* group, and *S. mitis* were accurately identified, as was *V. parvula*, a representative of the purple complex. *E. Corrodens* from the green complex was present in seven mock communities but detected in ten, resulting in three false positives. *Capnocytophaga* species *(gingivalis*, *ochracea*, *sputigena)* tested once as borderline positive though not present in the mock communities (Table 4).

### micro-IDent®plus11

*micro-IDent®plus11* correctly identified five bacteria present in the mock communities. *A. actinomycetemcomitans* serotype b, two representatives of the orange complex, *F. nucleatum* and *P. micra*, one representative of the yellow complex*, C. rectus/showae*, and one of the green complex, *E. corrodens*, were correctly detected. None of the other six bacteria covered by *micro-IDent®plus11* showed up as false positives (Table 4).

### Carpegen® Perio Diagnostik

Carpegen® Perio Diagnostik covers six periodontopathogens, three of which were included in the mock communities. Correct positive and negative results were obtained for *A. actinomycetemcomitans* serotype b, *F. nucleatum* and *P. intermedia* (both orange complex). None of the three other bacteria covered by *Carpegen® Perio Diagnostik* showed up as false positives (Table 4).

### PCR primers

PCR primers correctly identified three of the six bacteria included in the mock communities: *A. actinomycetemcomitans* serotype b, *C. rectus/showae*, and *S. mutans* (both yellow complex). Within the orange complex, *P. intermedia* was wrongly detected twice, rendering false positives in both evaluations for this bacterium (see above). *F. nucleatum* present in the mock communities was not detected in any of the seven positive samples. The tested primer pairs were shown to bind specifically to the *F. nucleatum* subspecies *vincentii* and *fusiforme* described in the original publications. The strain used in the mock communities was, in contrast, subspecies *nucleatum. E. corrodens* (green complex) was detected three times though not present in the mock communities. Other bacteria not included in the 14 mock communities were detected incorrectly: *P. gingivalis* (*n* = 6), *T. denticola* (*n* = 3), and *L. fermentum* (*n* = 10) (Table 4). Under the PCR conditions described in the original publications, unspecific bands could be detected on agarose gel for *T. forsythia*. Only samples showing bands with the accurate length were considered positive.

### Statistical agreement

When applied, testing resulted in exact Kappa agreement of statistical significance for all but one bacterium included in the commercial kits: *F. nucleatum*, *P. nigrescens*, *P. micra*, *C. rectus/showae*, *Streptococcus mitis group* and *S. mutans* as part of the *Streptococcus gordonii group*, A. *actinomycetemcomitans* serotype b and *V. parvula*, with a corresponding sensitivity of 100 % and a specificity of 100 %. The exception was *E. corrodens*, for which moderate agreement (*κ* = 0.57, *p* = 0.07) was reached with ParoCheck®Kit 20 and PCR primers, resulting in a sensitivity of 100 % and a specificity of 57 %. When analyzed using PCR primers, false positives were rendered for *P. intermedia* in both rounds of evaluation with a specificity of 86 %, while the purported false negatives for *F. nucleatum* revealed no sensitivity at all for this species (see discussion and Table 5).

**Table 5:**
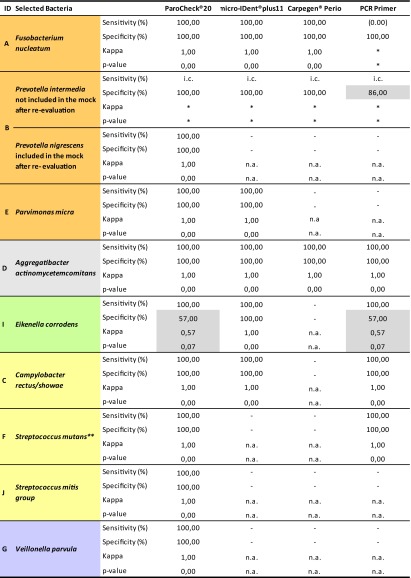
Agreement, sensitivity, and specificity of test kits and PCR primers (mock community as reference standard)

Interpretation of Kappa: <0.0 poor (no agreement), 0.0–0.2 slight agreement, 0.21–0.4 fair agreement, 0.41–0.6 moderate agreement, 0.61–0.8 substantial agreement, 0.81–1.0 almost perfect agreement

*n.a.* not applicable (not included in the kit), *i.c.* incalculable (not included in reference standard)

*All samples rendered negative results

**Included in *Strepotococcus gordonii* group

## Discussion

More than 700 species are reported to contribute to oral bacteria diversity [28]. When recent next-generation sequencing studies on oral microbiomes are considered, estimates of the number of species-level phylotypes range to as much as 10,000 when different sampling sites and pooled data from healthy and diseased individuals are taken into account [6, 13, 29, 30]. While the microbiome picture is expected to get even bigger as a result of refined methods, to date only 20 bacterial species have found their way into marketed periodiagnostics. Over the last two decades, methods based on PCR and cloning strategies have replaced time-consuming culturing procedures and produced commercial kits that are easy to use in dental practice. Even though the broadened view necessitated taxonomies to be modified in time, the species routinely tested in daily practice still correspond to the microbial complexes described by Socransky [17, 31]. In the present study, three commercial kits and formerly published PCR primers were tested for their accuracy in detecting *A. actinomycetemcomitans* serotype b and representatives of the orange, green, yellow, and purple complexes using mock communities as a reference standard. This approach overcomes some limitations to culture techniques traditionally used as gold standard. In our study the mock communities were used to verify test instruments available for outcome measurement in clinical and scientific settings. Scientific evidence, though contradictory, tends to support the use of systemic antibiotics and/or local antimicrobials as adjuncts to periodontal treatment [32–35]. Clear guidelines and distinct protocols are still lacking. Therapy decisions are based on interventional studies and in vitro experiments [36–39]. Commercially available kits and PCR primers are currently used in periodontal risk assessment, in monitoring therapeutic success and as an aid in choosing appropriate antibiotics [40–48]. Therefore, their accuracy and also the relevance of single representatives should be considered prior to use. In this way, the inherent bias of a test system selected to investigate a given issue can be minimized.

### *Fusobacterium nucleatum*

*Fusobacterium nucleatum,* a gram-negative obligate anaerobic rod, plays a major role in periodontal biofilm formation as a bridge between early and late colonizers [49]. Fluorescence in situ hybridization (FISH) shows *F. nucleatum* in only 12-h-old biofilms [50]. The species is divided into four subspecies: *nucleatum*, *polymorphum*, *animalis*, and *fusiforme*. A fifth subspecies, *vincentii*, was recently reclassified and now belongs to the subspecies *fusiforme* [51]. Our mock communities contained *F. nucleatum* subsp. *nucleatum*. While all three kits were accurate without exception, the strain was not detected using the PCR primers. These results underscore the primers’ high specificity for the subspecies *fusiforme*, as indicated in the original publications [27]. Jervøe-Storm et al. [8] and Verner et al. [52] showed that, compared with the cultivation method, detection reliability using real-time PCR varies from pathogen to pathogen. In their studies, *F. nucleatum* could not be distinguished from close relatives in the culturing experiments, yielding poor agreement for this species. The use of well-defined mock communities and the fact that commercial kits do not differentiate among subspecies explain their consistent agreement in our study.

### *Prevotella intermedia*

*Prevotella intermedia* (formerly *Bacteroides intermedius*) is a gram-negative obligate anaerobic rod that is often isolated from oral cavities. Closely related species, *P. intermedia* and *P. nigrescens*, can pose a challenge to differentiation, as reported by previous culture experiments [8, 25, 52]. Since only *P. intermedia* is associated with periodontal disease, reliable differentiation is vitally important [53].

### *Parvimonas micra*

*Parvimonas micra* (previously *Peptostreptococcus micros* or *Micromonas micros*) occurs in pairs and short chains as gram-positive obligate anaerobic cocci. As an orange complex periodontopathogen, it is associated with most types of oral infection such as periodontitis, endodontic and acute dentoalveolar infections, pericoronitis, and advanced dental caries [54, 55]. It also has been isolated from non-oral diseases, primarily from soft-tissue abscesses and bite wounds but also in the course of spondylodiscitis [56]. Recently, in a case control study, *P. micra* was found to be the only microbial predictor of periodontal parameters. *P. micra* is regarded as an important periodontal pathogen warranting more attention [57].

### *Aggregatibacter actinomycetemcomitans*

Compared with other periodontopathogens, *A. actinomycetemcomitans* (previously *Actinobacillus actinomycetemcomitans*) is reported to be less prevalent and more heterogeneous. The genetic diversity and epidemiological distribution of *A. Actinomycetemcomitans*is strains has been frequently studied [58–62]. As a gram-negative facultative anaerobic subgingival biofilm former, some strains are clearly associated with periodontal disease [25, 63, 64]. While serotype c is commensal in healthy populations, serotype a and b strains are associated with severe periodontitis. *A. actinomycetemcomitans* strains exhibit a wide range of variability with regard to leukotoxin production. The specific JP2 clone, a highly leukotoxic strain, plays an important role in the development of aggressive periodontitis in certain populations [65–68]. Serotype d and e are rare in all populations. However, there is still a lack of evidence about the infectious etiology of destructive periodontal disease [69]. In the present study, *A. actinomycetemcomitans* serotype b was included as reference strain in the mock community. It was detected accurately in all cases by the kits and PCR primers. Thus, when used as a reference standard, our mock communities result in a higher specificity for this species than has ever been previously published. The reported specificity levels range from 10 to 90 % when real-time PCR was compared with bacterial cultures as the reference standard [70]. However, the kits and primer under investigation claim to cover serotype a, b, and c and are therefore unable to distinguish between the highly pathogenic genotype b and the non-pathogenic genotype c. Future studies are needed to test more highly specific primers in screening for *A. Actinomycetemcomitans* strains of diverse pathogenicity.

### *Eikenella corrodens*

*Eikenella corrodens* belongs to the *Neisseriaceae* family and is frequently found in the oral cavity. It is one of the HACEK bacteria (including: *Haemophilus* species, *Aggregatibacter* species, *Cardiobacterium hominis*, *Eikenella corrodens*, and *Kingella* species). This group of gram-negative facultative anaerobic bacteria frequently colonizes the oropharynx. *E. corrodens* has long been recognized as a cause of infective endocarditis [71]. Additionally, it has been implicated as an oral pathogen in Socransky’s green complex. The accentuation of the green complex in periodontally diseased pockets needs to be considered in antibiotic therapy. The false positives obtained from ParoCheck®Kit 20 and PCR primers when *Neisseria subflava*—also belonging to the *Neisseriaceae* family—was present in the mock communities, indicates a specificity problem.

### *Capnocytophaga canimorsus*

*Capnocytophaga* spp., gram-negative facultative anaerobes, are present in habitats of the human oral cavity and some are associated with periodontitis. They belong to Sokransky’s green complex. To test specificity, *C. canimorsus* was added to the mock communities. This strain is usually found in the microbiota of canines [72]. The one marginally positive result found with ParoCheck®Kit 20 may reflect an unspecific primer attachment due to the presence of *C. canimorsus* in the respective samples.

### Campylobacter rectus/showae

*Campylobacter showae* strains in the human gingival crevices were first characterized and distinguished from *C. rectus* by Etoh et al. [73]. In general, *C. showae* strains are catalase-positive and resistant to nalidixic acid, while *C. rectus* strains are catalase-negative and sensitive to that antibiotic. Macuch and Tanner [74] suggested that *C. showae* may be associated with periodontal disease and confirmed the relationship between *C. rectus* and diseased subgingival sites. *C. rectus* can be used as a marker for periodontal disease progression [75]. Specificity testing against other more highly commensal strains of this group, such as *C. gracilis* and *C. concisus*, was not part of the present study, but such strains could be included in a future mock community.

### *Streptococcus mitis* group and *Streptococcus mutans*

The *Streptococcus mitis* group (SMG) belongs to the viridans *Streptococci.* These ubiquitous initial colonizers constitute a majority of the cultivable bacteria found in dental plaque [76]. The *Streptococcus mitis* group comprises: *S. mitis*, *S. sanguinis*, *S. parasanguinis*, *S. gordonii*, *S. oralis*, *S. cristatus*, *S. infantis*, *S. peroris*, *S. pneumoniae*, and *S. pseudopneumoniae*. ParoCheck®Kit 20 was the only kit to include *S. mitis*. The kit promises detection of the entire *S. mitis* group as well as *S. gordonii*, which is referred to as the *Streptococcus gordonii* group and also comprises *S. mutans*. ParoCheck®Kit 20 detected *S. mutans* in the *Streptococcus gordonii* group with 100 % specificity. *S. mutans* primers developed by Chen et al. also showed comparable specificity [26]. micro-IDent®plus11 and Carpegen® Perio Diagnostik exclude *S. mutans* from analysis, regarding it as being related to caries and not to periodontal desease. As *Streptococcus* spp. were shown to co-aggregate in vivo with *Veillonella* spp., *Fusobacterium nucleatum* and *Actinomyces naeslundi,* they can be assumed to play a relevant role in periodontopathic biofilm [77]. Elevated *S. mutans* levels appear to correlate directly with increased severity of periodontal disease among untreated elderly patients [78]. The subgingival area is a microbial habitat not only for periopathogens but also for mutans streptococci, indicating a disturbed micro environment of the oral cavity [9]. They also may be of importance in the development of root caries in periodontitis patients [79]. However, the detection of *S. mutans* in subgingival plaque, while having no consequence for treating periodontal disease, might be useful in answering specific research questions. Future studies should provide insight not only into the diagnostic and therapeutic value of periodontal test kits and primers but also into their cost-effective application as preventive measures.

### *Veillonella parvula*

*Veillonella parvula*, an anaerobic gram negative coccus and lactate utilizer, is almost always found in association with *Streptococci*. All are known as first colonizers on clean tooth surfaces in the human mouth. These highly prevalent representatives of the *Firmicutes* phylum are characteristic of the microbial community associated with common dental plaque and usually not associated with oral infections [31, 80]. Due to the great abundance of *Firmicutes* in oral biofilm, relative changes can be observed easily and might deliver valuable information for monitoring oral health and disease. In our study, *V. parvula* was only included in ParoCheck®Kit 20, which revealed accurate test results for all samples.

### Commercial kits versus laboratory-developed PCR primers

Despite some limitations, commercial kits proved to be useful for obtaining information about the state, progression, and therapeutic outcome of periodontal disease [36, 81, 82]. Non-invasive subgingival paper-point insertion allows DNA to be collected at chairside within seconds. Paper points are packed in sterile tubes and sent to specialized laboratories. DNA extraction, preparation, and analysis are then performed within 3 h. When compared with laboratory-developed primers, the major advantage of the commercial kits is their improved accuracy. In the case of *A. actinomycetemcomitans*, however, it would be helpful if detection was limited to the highly pathogenic genotype b, without detecting the non-pathogenic genotype c. In the routine diagnostic laboratory, it is preferable to use an IVD assay that bears the CE label and/or is approved or cleared by the FDA. It is also advisable to employ such kits in clinical research [40–48]. In this study, the protocol for bacterial suspension and DNA extraction was aimed to clearly exceed the detection limit of 10E3 given for the commercial kits. Further studies with a variation in study design are needed to test the performance levels of the kits at the detection limit. Carpegen® Perio Diagnostik in particular allows bacterial counts based on real-time PCR, thus enabling quantitative comparisons. The present study, however, was restricted to the specific detection of selected pathogenic bacteria and excluded quantification. The kit detects six representatives from the red, orange, and green complexes, all of them highly associated with periodontal disease. The other two kits tested in the present study, in contrast, allow only semi-quantitative analyses based on a DNA chip or DNA strip technology. On the other hand, the latter cover a wider range of bacterial species. micro-IDent®plus11 additionally includes representatives of the yellow complex, while ParoCheck®Kit 20 also covers species from the purple and blue complexes. While these additions do not necessarily improve diagnostic and therapeutic outcomes, they can provide additional value in clinical investigations of oral biofilm from healthy and deceased subjects.

Compared with the three tested commercial kits, the previously published PCR primers showed less agreement for the mock communities. In the case of *F. nucleatum*, the “in-house” primers were obviously more specific to subtypes of the respective periopathogen, resulting in “pseudo”-negative results. Primers were selected with the intention of embedding them in microbiological screening to obtain scientific insights useful in the risk assessment of periodontal disease. The results in this study apply primarily to the strains included in the mock community. However, some bacteria not included in the mock communities, such as *P. gingivalis*, *T. denticola*, *Capnocytophaga*, and *Lactobacillus fermentum*, were nonetheless detected by primers. Due to the relevance for screening procedures, such results were reported as false positives, considering that no false negatives can be reported for a species or strain that is not part of the reference standard. These bacteria should not have been detected at all, regardless of the primers’ specificity level. Primer pairs as tested in this study consequently present a twofold specificity problem: they either (a) seem to be very specific to subtypes/strains and are therefore limited to the purposes stated in the particular study or (b) their specificity is so low that bacteria not present are detected. These incorrect results highlight the difficulties in primer development. Applied molecular assays for primer composition and variable PCR conditions as extensively discussed elsewhere represent possible sources of error. We suggest validating primer pairs through prior testing using mock communities. Such should comprise a great variety of bacterial species, including species not present in the oral cavity but elsewhere in (and on) the body to assure high accuracy. Though more error-prone, the big advantage of “in house” primers is their taxonomic scope, which can be adjusted to be highly specific by designing them in line with various research or diagnostic purposes. Regardless of whether “homemade” or “instant,” it is obvious that the more specific a primer is, the more accurately it will be able to resolve bacterial lineages.

### Mock communities

The mock communities used in the present study were selected from the strains of bacteria that were available at the research laboratory. A limitation of this study is the fact that the bacteria selected did not include the full array of strains potentially covered by the tested kits or primers. However, *A. actinomycetemcomitans* serotype b and yet little investigated representatives of the orange, purple, green, and yellow complexes as described by Socransky et al. could be tested in this study, while bacteria from the red complex have been studied extensively elsewhere [18–21]. Strains were chosen from among those in the laboratory stock that corresponded as closely as possible to the detection profile claimed for the three commercial kits and the primers. In addition, bacteria not covered by the kits or primers were also included in order to test for general specificity. The goal in randomly mixing the bacteria among the mock communities was to ensure that the available strains were present in a variety of combinations and in varying proportions within the communities. Neither the dimension of pathogenicity of the selected strains was a criterion for inclusion nor was there a focus on increasing the number of detectable species. Rather, this study was intended as the first one of its kind, critically examining the accuracy of three commercially available test kits and published primers against mock communities as a potential formal reference standard. As the study shows, the approach can be easily standardized and adapted for more comprehensive criteria to be applied in future studies, such as equal distribution or a wider assortment of strains.

## Conclusion

Presenting a first comparison of three commercial kits and laboratory-developed primer pairs with regard to effectiveness in detecting periodontopathogens, we confirm that the commercial kits used in this study are reliable tools for periodontal diagnostics and therapy. Whereas the detection profile of kits is fixed at a general specificity level, the design of primers can be adjusted to differentiate between highly specific strains. In-house primers are more error-prone and should be carefully designed and tested prior to use. We suggest bacterial mock communities be established as a reference standard for any similar testing of kits and primers.
